# Defining Requirements and Related Methods for Designing Sensorized Garments

**DOI:** 10.3390/s16060769

**Published:** 2016-05-26

**Authors:** Giuseppe Andreoni, Carlo Emilio Standoli, Paolo Perego

**Affiliations:** Design Department, Politecnico di Milano, via G. Durando 38/A, 20158 Milan, Italy; carloemilio.standoli@polimi.it (C.E.S.); paolo.perego@polimi.it (P.P.)

**Keywords:** wearable systems, textile sensors, ergonomics, human factors, sensorized clothes, smart textiles, sensor design

## Abstract

Designing smart garments has strong interdisciplinary implications, specifically related to user and technical requirements, but also because of the very different applications they have: medicine, sport and fitness, lifestyle monitoring, workplace and job conditions analysis, *etc.* This paper aims to discuss some user, textile, and technical issues to be faced in sensorized clothes development. In relation to the user, the main requirements are anthropometric, gender-related, and aesthetical. In terms of these requirements, the user’s age, the target application, and fashion trends cannot be ignored, because they determine the compliance with the wearable system. Regarding textile requirements, functional factors—also influencing user comfort—are elasticity and washability, while more technical properties are the stability of the chemical agents’ effects for preserving the sensors’ efficacy and reliability, and assuring the proper duration of the product for the complete life cycle. From the technical side, the physiological issues are the most important: skin conductance, tolerance, irritation, and the effect of sweat and perspiration are key factors for reliable sensing. Other technical features such as battery size and duration, and the form factor of the sensor collector, should be considered, as they affect aesthetical requirements, which have proven to be crucial, as well as comfort and wearability.

## 1. Introduction

From the technological and societal point of view, the 2015–2025 decade has been identified as the “Wearable Era” [1,2,3]. This decade is characterized by the implementation and large diffusion of new miniaturized and wearable products supporting innovative sensing and feedback functions and related services with potentially enormous impacts on our lives. Most of these systems collect data and information from our body and also support a basic data processing to provide the user with immediate feedback about his/her status and/or lifestyle. For this reason, they are often considered health-related devices. Wearable Health Systems (WHS) or Wearable Biomedical Systems (WBS) are integrated systems on body-worn platforms such as wrist-worn devices or even biomedical clothes, offering pervasive solutions for continuous health status monitoring though non-invasive biomedical, biochemical, and physical measurements [4].

**Figure 1:**
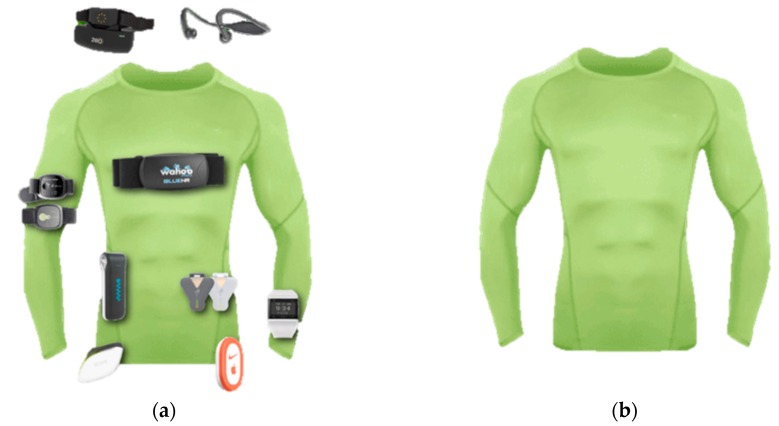
The comparison of the two generations of Wearable Health Systems (WHS). (**a**) Wearables 1.0: the garments and other accessories are the supporting platform of a set of devices for monitoring human functions; (**b**) Wearables 2.0: sensors and related electronics components and integrated into the garments (adapted from [5]).

Thanks to their continuous miniaturization, they support a non-intrusive measurement of user parameters. The non-intrusive monitoring paradigm is when the user forgets the ongoing process. This means that, even if wearing these devices, he/she is not aware of the continuous monitoring, but he/she can retrieve data and related information whenever he/she wants, thanks to mobile technology. Several fields of application are under exploration and exploitation, and among them the main ones are medicine, life style monitoring, and sport analysis (including, in this sector, fitness). Currently, we are undertaking the realization and diffusion of the first generation of these systems. They are small devices that have usually one or limited sensors, and a plurality of devices to be worn is needed for a comprehensive approach (Figure 1a). Research and innovation is facing the evolution of these systems, where our garments represent the worn platform in which to embed sensors and processing units. This technological advance makes use of smart fabrics and yarns to empower our natural interface with the external world, *i.e.*, our clothes, to provide new and high-level functions. This represents the 2.0 generation of WHS (Figure 1b).

The application of WBS in medicine would offer the opportunity to monitor patients easily and over extensive periods of time, thus implementing the telemedicine definition: to offer affordable and interactive healthcare, anyplace, anytime to anyone. This will allow the implementation of the strategy of Western countries of offering high quality services with reduced healthcare costs, which is the ultimate requirement and the future of healthcare. Clinical applications are probably most critical due to their requirements in terms of signal quality and device certification. However, by matching these requirements, it is possible to simplify the systems for high level applications turning them into reliable solutions for lifestyle or sport monitoring functions [1,2,3,4,5,6,7,8,9,10,11,12,13,14].

Designing and implementing wearable systems and specifically sensing garments might seem like a simple activity; however, a plurality of disciplines is needed to interact and integrate to develop the right solution. Several requirements at different levels have to be taken into account. Sometimes, even obvious elements can become critical in an integrated approach; for example, washability is an obvious requirement for a garment, but this can irreversibly deteriorate an integrated sensor. A simplified list of requirements to follow could be as follows: sensors and actuators, materials, communication (usually wireless), power supply and management, processing techniques (onboard/on external devices, off line/real-time), Graphical User Interfaces (GUIs), algorithms for signal processing, connectors, washability and the related stability of sensors, body positions and wearability (tasks, sensors, anthropometry, *etc.*), elasticity, and adherence to the supporting platform (garments or belts, an adhesive patch, or other).

Here, the authors discuss the most significant aspects of designing a sensing wearable platform, specifically in relation to the implementation and integration of textile sensors or other sensors for physiological signal monitoring. The discussion is on two levels: the technology requirements, focusing in particular on the sensor design and materials, and the design specifications, where specific focus on anthropometry and garment features are presented.

## 2. Materials and Methods

When designing wearable systems, two main sets of specifications can be outlined: the technological features and the design requirements. The main issues related to technology are the identification of the sensing principle and the corresponding sensor implementation, the selection of the right materials and their properties, data storage, data processing and related algorithms, data transmission (bit rate, distance, *etc.*), and the amount of power to be supplied in accordance with the desired functioning time, processing, *etc*. These are very technical issues but strongly and mutually influence the design issues. In fact, the design factors include the physical features of the device such as shape, the form factor, the softness/rigidness of the case and related materials, and other design-related issues in relation to anthropometry and gender, GUIs, positions on the body and/or into the garments, wearability, elasticity, and adherence to the body fixing element or of the garments.

### 2.1. Technological Specifications of Wearable Systems

The main goal of a wearable system is to collect information about the user wearing it. These data could be of a different nature and level of importance (the highest level and the corresponding required quality is obviously needed for medical applications/services). Usually, non-invasive biosignals are measured and stored in the systems or transmitted to a body gateway. These biosignals are measurable and can be collected on the body surface (usually the skin) through specific sensors. The first requirement is to define the physical nature of the signal to be measured and the corresponding sensor [15]. The main categories of signals are:

- bioelectric signals, such as Electro-Cardio-Gram (ECG), Electro-Myo-Gram (EMG), Electro-Encephalo-Gram (EEG), Electro-Oculo-Gram (EOG), Electro-Neuro-Gram (ENG), and others;
- thermal parameters, such as body temperature in specific points and its map on a specific portion of the body surface;
- mechanical measurements, such as the kinematics of body segments and the pressure at the human/object interface (e.g., the human back and a mattress);
- optical signals, such as the SpO_2_ assessment and other NIRS measurements;
- chemical analyses, such as the determination of the composition of the sweat and that of the inhaled/exhaled air.

**Figure 2:**
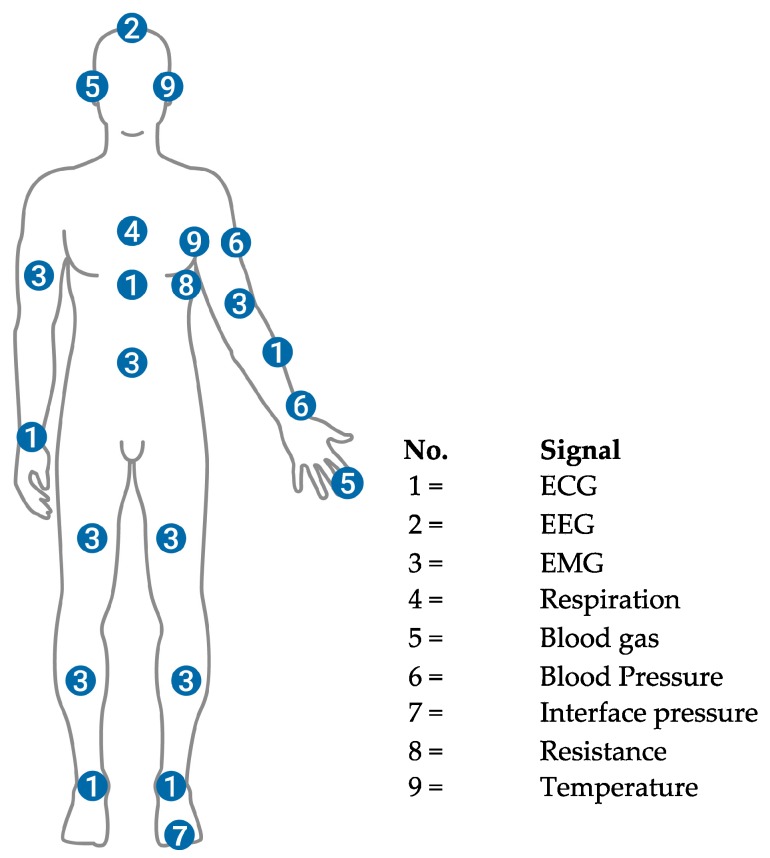
A simplified map of the bio-signals to be measured from the human body through wearables systems and the corresponding sensing point. ECG: Electro-Cardio-Gram, EMG: Electro-Myo-Gram, EEG: Electro-Encephalo-Gram.

Table 1 describes the sensor to be used in the measurement of each bio-signal and its typology, while a simplified map of the sensing point to the body for each signal is reported in Figure 2.

The basic sensors to measure bioelectric signals are electrodes. The standard solution is represented by adhesive, non-polarized, silver/silver chloride (Ag/AgCl) electrodes [15,16], which are small discs with a stainless male snap button in their center and that are directly placed on the body in specific positions according to the measurement setup and requirements. The snap is reached by a wire that, on the other side, is connected to the measuring device. In the vision of the 2.0 wearable generation, these electrodes are transformed into textile electrodes (sometimes also called textrodes). They can be implemented by knitting or weaving or by integrating the electrically conductive yarns with other textile techniques (e.g., embroidering) to create sensing areas in specific positions of the clothes. The other essential requirement is that these sensing zones have to be in contact with the skin to assure the proper signal measurement. They are a category of polarized electrodes. In general, non-polarized electrodes are better than polarized electrodes in terms of their rejection of motion artifacts and their response to defibrillation currents.

Different solutions are available to implement textrodes, specifically in terms of textile yarns with electrical properties; these yarns can be classified into two main categories [4,8,16]:

(a) metal yarns, *i.e.*, yarns containing conductive fibers such as stainless steel, copper, and silver mixed with natural or synthetic fibers;
(b) yarns containing electro-conductive fibers such as polymeric or carbon-coated threads.

Table 2 summarizes the materials used in textrodes with pros and cons.

The adoption of a specific material (and the yarn-related properties) is also a unique choice for the textile solution in terms of production technique and final characteristics of the sensing garment. In fact, the selection of the fibers determines elasticity, washability, and the stability of the sensor properties to the chemical agents used for cleaning. An improper stability could affect sensors’ efficacy and reliability, and this factor is also crucial in the definition of the proper duration of the product and its life cycle.

**Figure 3:**
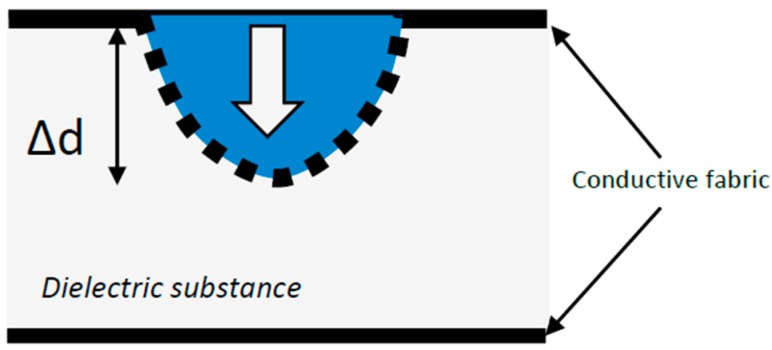
A model of capacitance sensor implemented with two layers of conductive fabric and a spacer that could be a 3D textile.

**Figure 4:**
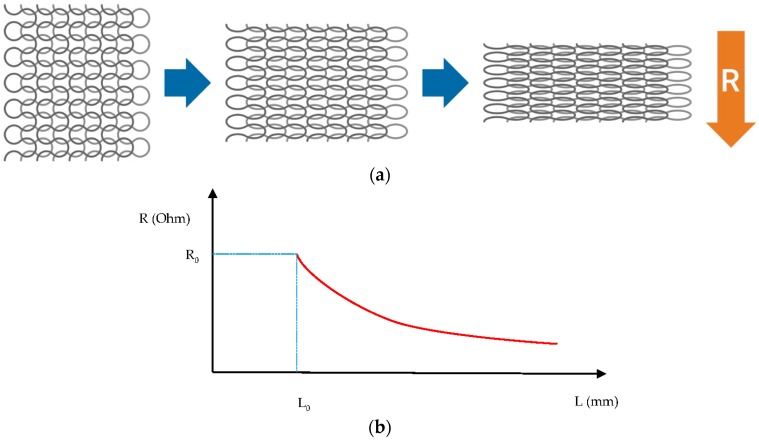
A model of a textile strain gauge and the related description. (**a**) The modification of the yarn configuration inside the fabric while stretching it; a wider superficial contact area is obtained among the different conductive yarns, thus resulting in a decrease in the electrical resistance. (**b**) The typical graphical representation of the mathematical equation that expresses the relationship between elongation and electrical resistance of the textile strain gauge.

The choice of the material is important in order to define the proper measurement set-up and system. For example, a capacitance sensor can be built using dielectric materials (Figure 3). In this case, capacitance increases with applied force. Another type of pressure sensor is a piezo-resistive element. Instead of using a dielectric material, it is possible to insert a piezo-resistive substance between the two textile plates/contacts. Therefore, force produces a deformation of the material and its structure or conductive content. Usually, resistance decreases with the increase in the stimulation signal (Figure 4), but this effect depends on the used material or its construction (e.g., yarn directions with respect to the elongation direction that can produce a positive or negative coefficient into the resistance-strain function that corresponds to the gauge factor). Therefore, this sensor measures the applied force on its surface (which can be also used for measuring the interface pressure distribution on a wider area in a configuration of a mat of sensors) and requires a very different circuitry in accordance to the sensing principle [17,18,19]. Applications of these textile pressure sensors are multifold. One example is provided by an innovative system for sleep monitoring that integrates some of them into a sensorized blanket; another product is aimed at monitoring walking or running performance and/or foot disorders by putting these textile capacitive sensors into insoles or socks. This system is capable of measuring—nay, of estimating, because they are related only to some specific points of the foot—the pressure points under the foot during gait or exercise. Other solutions or concepts that integrate the pressure sensors into monitoring systems are dedicated to posture analysis, for example, by being used in sensorized seat cushions (or simply their coverings) [17,18,19,20,21].

The material that the sensors are made of also influences the property of a skin-sensor contact and interface [8,16,17,18,19]. From the operational point of view, proper sensing requires good conductance and a stable skin-sensor contact. Standard silver/silver chloride adhesive electrodes use Ag/AgCl gel to establish a good conductive contact with the skin, consequently improving the output signal. However, many patients experience a certain discomfort since this gel may cause skin irritation and softening, especially in the case of prolonged application. These inconveniences impose restrictions on the use of this kind of electrode for long-term monitoring.

For textrodes, some preparations are required to have good signal quality: skin cleaning, scrubbing the external layer of dead skin cells, and removing the presence of hair, especially in male subjects. Textrodes are usually dry sensors, but they are capable of receiving, and sometimes retaining, perspiration and sweat to increase local contact conductivity. In fact, in textile electrodes, the presence of sweat is demonstrated to be useful because it is a conductive solution, thus improving the conductivity of the electrical contact with the skin and the signal quality revealed through the electrode [8,16,17,22]. Sometimes, before the perspiration effect arises, in order to have an immediate good sensing capability, wetting the textile electrode before wearing the smart garment could immediately increase the quality of the skin-electrode contact. In some garments, this effect could be obtained with the insertion of small sponges placed under the sensing surface (in a small pocket) or filling the space underneath the sensor with hydrophilic yarns; this process also produces a convex sensor surface that helps to increase the skin contact pressure. In this way, the sensor can stay in a more stable position on the skin itself [19]. Thanks to these design solutions and the effect obtained (usually after a few minutes) as a result of the local sweat (perspiration), electro-gel in textrodes was usually found to be unnecessary.

Indeed, textrodes also represent a possible solution to the limitations related to the other possible effects such as the production of cutaneous events due to the material in contact with the skin (tolerance and irritation, and/or reactions or modifications due to sweat and perspiration). One of the main advantages of textrodes is that they do not irritate the skin, even in prolonged application. In any case, these situations have to be properly investigated before the sensor implementation or application to subjects. In fact, not all the materials are suitable for textrodes implementation: carbon black has been shown to produce similar skin effects, while silver or stainless steel textile electrodes are safer [23].

Instead, the main disadvantage of textile electrodes (in particular with respect to the standard adhesive electrodes) is the unstable skin/electrode contact due to the non-adhesive surface contact. This means that a dedicated design of the garment and/or higher performances on the electronic circuit are required. For example, the analogue front-end of the sensing circuitry needs to be redesigned and optimized for better signal amplification and noise reduction [9,22].

In relation to the signal quality, in a previous work, we analyzed the comparison between standard electrodes and textrodes [24]. We analyzed the reliability of textile electrodes compared to standard adhesive electrodes using a two-channel portable electro-cardiograph in two conditions: (a) *in vitro*, by using a ECG signal generator; and (b) *in vivo*, registering the signals of 10 subjects during a set of standardized activities of daily living, such as walking, standing and sitting down, and stair climbing. The experimental setup consisted of a very close set of four electrodes paired in twos (1 was textile and 1 was a traditional adhesive Ag/AgCl electrode) to verify that the quality of the signal and the computed parameter in time and frequency were comparable. Results demonstrated a very good reliability. 

**Figure 5:**
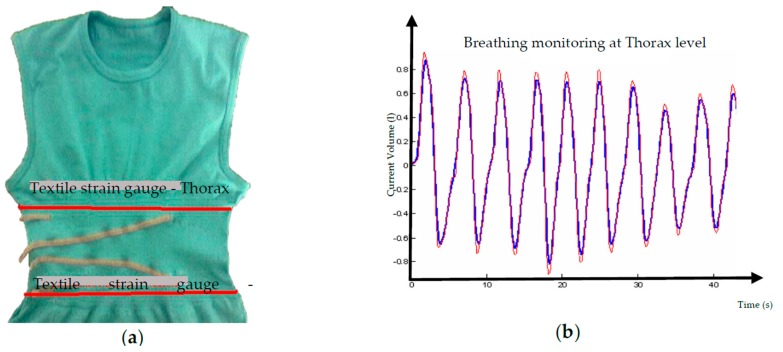
An example of an application of textile strain gauges in the monitoring of respiratory acts and related parameters. (**a**) A sensorized T-shirt for monitoring respiration through textile strain gauges at thorax and abdominal level; (**b**) The comparison of the signals from the textile sensor and the pneumo-tachograph (gold standard technique); the blue line shows the gold standard data measured during normal breathing at resting in sitting posture, and the red line shows the textile strain gauge signal in the same condition. A ~5% air volume overestimation at peaks is shown.

Textile structures containing electro-conductive material could be used as textile strain gauges when they are carefully engineered and characterized. A strain gauge exhibits a percentage change in resistance that is directly proportional to the applied strain (Figure 4). In this case, they are suitable for measuring mechanically induced elongations (from motion of body structures). With proper calibration, they can provide satisfactory accuracy in the measurements of breathing parameters or joint movements (Figure 5). The following equations describe the process:

S = dL/L_0_, and (1)

dR/R_0_ = S_g_·S, (2)

where S is the strain, L is the actual length, L_0_ is the length at rest, R is the actual electrical resistance of the textile elastic element, R_0_ is the electrical resistance of the textile elastic element at rest, and S_g_ is the gauge factor, *i.e.*, the coefficient to convert the strain to dR/R_0_.

As a possible solution for sensor characterization, we also defined an experimental setup to identify the relationship between the electrical resistance and the sensor elongation. The experimental setup usually pairs a displacement measurement system with a four-wire resistance measurement circuit, as described in detail in [25]. In that case, a set of two realizations of textile strain gauges was tested obtaining the calibration elongation-resistance. The curve increased exponentially according to the different yarn distribution with respect to the example in Figure 4a. Thus, through characterization, the elongation textile sensor can be used for reliable signal measurements. Figure 5 displays the experience of using these textile strain gauge sensors to measure breathing parameters. We have applied two textile strain gauges along the circumference of the human trunk—one at the hearth level, and one on correspondence of the abdominal compartment. Simultaneously with the textile sensors monitoring, the pneumo-tachograph (*i.e.*, the gold standard technique) recorded breathing parameters. As shown in Figure 5b, the correspondence of the two signals—the temporal parameters (breathing rate, inspiration time, expiration time)—perfectly match, while the computation of the current volume suffers from some underestimation (average difference −6%) in the peaks. This demonstrates how a simple textile structure in the garment could be very useful and reliable for monitoring this vital sign. By integrating this technique with textrodes for ECG measurement and adding an Inertial Movement Unit (IMU) (for body movement measurement, *i.e.*, actigraphy) into the electronic frontend for signal acquisition, a simple but almost complete compact system for basic monitoring was obtained. This solution can be easily applied to medical and sport monitoring applications [11,12,13,23].

### 2.2. Design Specifications of Wearable Systems

A basic requirement for the measurement process is a good, stable contact between the body and the sensor for the proper sensing time. Ideally, in the case of monitoring, it should be in continuous close contact with the body and without movements of the sensors over the skin. This last event produces artifacts definitely corrupting the signal pattern and quality. For this reason, anthropometric factors and gender considerations, that are also related to body sizes and shapes and have implications on user preferences, should be among the first ones to be addressed in terms of the design of wearable monitoring systems.

Anthropometry is the measurement of physical characteristics of the human body and their differences in relation to age, gender, lifestyle, and ethnicity as inter-subject variability factors, or even time. For example, stature varies in the same subject with time and according to different phenomena [26]. In a short time, the height of people usually follows a circadian cycle: subjects are about 0.7%–1% higher in the morning than in the evening. Over long periods, for example, stature varies according to growth or the ageing processes: there is a large increase in stature during growth in childhood, and a moderate decrease in the late adult age. Also, living in external environments could influence anthropometry (and therefore garment design): e.g., in microgravity (that is an extreme condition) when a stature increase of about 4% is reached and kept after a few days of permanence in space, as well as the fluid shift produces variations of volumes of different body districts. Gender issues are quite obvious: men and women have different requirements not only for their different dimensions, but for shape, fit, and physiology, as well as for the daily task these users undertake.

**Figure 6:**
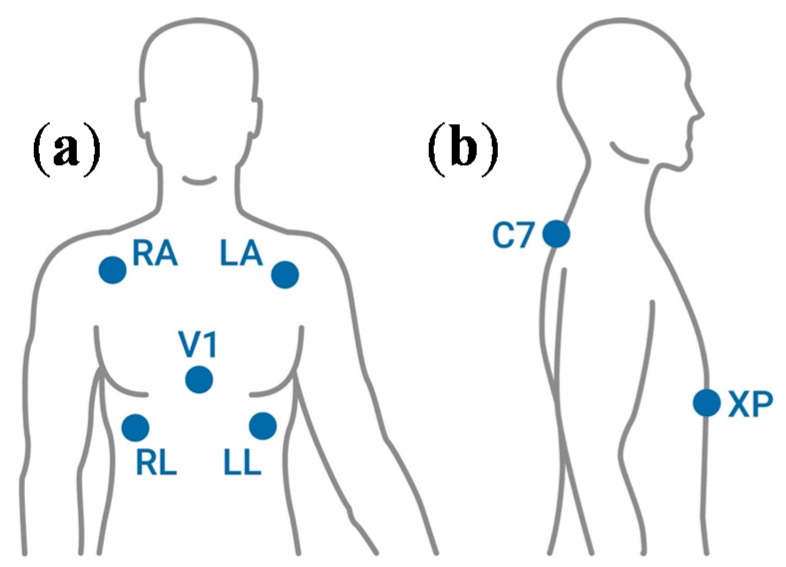
The electrode setup for measuring heart electrical activity. (**a**) The standard sensor positions for Electro-Cardio-Gram (ECG) measurements in the reduced five-lead configuration; (**b**) Transversal ECG sensors setup to avoid pectoralis muscles artifacts (C7 = in correspondence with the 7th cervical vertebra, XP = in correspondence of the xiphoid process).

**Figure 7:**
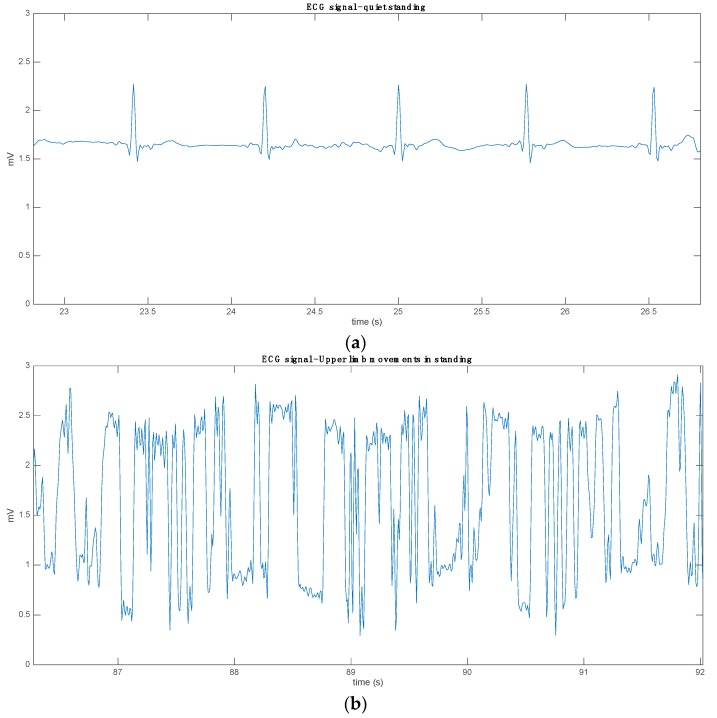
The effect of movement artifacts on bioelectrical signal recordings: an example of the different signal quality with the “standard” ECG lead implemented by two sensors on the left and right costal chest and in correspondence with the 10th rib (RL and LL in Figure 6). (**a**) 4 s of the typical ECG pattern recorded during quiet activity in sitting posture (typing). (**b**) A corrupted 4-s ECG signal with no recognizable peaks during upper limb movements (frontal abduction/adduction) in the same posture.

The functional aspect is crucial for identifying the best places for sensors for different applications. This is crucial, for example, in sports activities, where the contribution of the upper limbs and their synergic, antagonistic, or synchronized movement could produce different signal artifacts. For example, the most used electrode configuration for heart electrical activity monitoring is the two-sensor setup: it requires two electrodes placed on the right and on the left side of the chest, approximately in correspondence to the 10th rib (Figure 6). When the subject performs wide and/or strong upper limb movements, EMG artifacts are produced by pectoral muscles. The EMG signal is superimposed to the electrical activity of the heart and this could significantly affect the quality and the reliability of the ECG signal recording. Thus, an equivalent but transversal lead (*i.e.*, bipolar electrode set-up) should be used to minimize the incidence of these artifacts (Figure 7); therefore, a specific design of the sensing garment is required.

A study and design of the proper elasticity of the garment at different levels of the trunk, and in specific body areas where the sensors are present, is needed. The specific and modulated elasticity in the different parts of the garment is needed to assure good sensor-skin contact and to minimize their movements over the skin during the recording [23]. This phenomenon, also known as a skin motion artifact in biomechanical analysis with passive markers and opto-electronic motion capture systems, applies to wearable monitoring as well, and its avoidance or minimization is crucial for a good signal quality for most of the measuring time. Again, this possible critical issue can be solved by keeping a close and stable contact of the sensors with the skin. This requirement is fundamental also in relation to the small value of the measurand, e.g., bioelectric signals having a peak-to-peak amplitude ranging from 0.5 mV up to 2 mV. An example of this is reported in Figure 7, which shows an ECG signal collected through textrodes. The signals are collected with the same positioning of the textile electrodes, but the only variable factor is the resting position of the upper limbs (Figure 7a). This is compared to the same setup, but with movement in the upper limbs in the horizontal plane at shoulder level (Figure 7b). In this second condition, it is clearly shown that the ECG pattern is completely corrupted by the artifacts. The sensorized T-shirt has high elasticity, and its size was greater than the one worn by the subject. All these factors, which may seem very simple to address, are equally crucial with respect to the technological factors in wearable applications.

For this reason, the study and the design of the garment is crucial for its elasticity and body fitting, in particular for obtaining a good adherence to the garment in the region of the cloth and of the body where the sensors are placed. Modulated elasticity is obtained by proper choice of elastic yarns and/or by garment design. Comfort and wearability of the garments itself are not to be forgotten at the same time. In fact, wearability is crucial for weak users (e.g., elderly, patient under rehabilitation, *etc*.) for autonomous operations and repeatable sensor inter-session repositioning to achieve a reliable monitoring. 

Finally, but with the same importance and priority, aesthetical requirements should address user preferences that are crucial for the acceptance and usability of the systems (garments, devices, *etc.*). This can determine the final success of the wearable monitoring approach. Design and aesthetics include the analysis of several factors including the user’s age, the target application, possible competitors, and fashion trends. The personal preferences are also particularly important because the sensing garments are usually the underwear, and there is a requirement that the sensors be in close contact with the skin. Thus, color, material (cotton *vs*. technical fabrics), fitting, and the choice of substituting or integrating it with one’s own underwear (this specifically applies to elderly women that often prefer a sensorized belt to be worn in parallel and in addition to their traditional bra, despite the availability of a new integrated solution) are fundamental aspects to be considered in the system design.

Gemperle *et al.* [27] analyzed the wearability requirements for hardware systems that have been formalized in a 13-point guideline here reported: (1) placement (where on the body it should go); (2) form language (defining the shape); (3) human movement (consider the dynamic structure); (4) proxemics (human perception of space); (5) sizing (for body size diversity); (6) attachment (fixing forms to the body); (7) containment (considering what’s inside the form); (8) weight (as its spread across the human body); (9) accessibility (physical access to the forms); (10) sensory interaction (for passive or active input); (11) thermal (issues of heat next to the body); (12) aesthetics (perceptual appropriateness); and (13) long-term use (effects on the body and mind).

## 3. Discussion

In the technology-based medicine era, the innovation and development of new technologies represent a winning strategy to create solutions for more accurate, personalized, and continuous healthcare services [1,2,3,4]. Personal Health Systems (PHS) were introduced in the late 1990s to support the generation of innovative healthcare services (e.g. remote ambulatory monitoring, home care, *etc.*) for a personalized medicine. PHS empower and put the individual citizen/patient at the center of the healthcare process. They empower citizens/patients because they allow them to have more responsibility in managing their own health thanks to the amount of data and information they can provide directly to the patients themselves and/or to their related care providers. The main benefits of PHS for citizens and health authorities are a significant improvement in the quality of care for the individuals themselves because continuous non-intrusive monitoring allows for a light process, often delivered at home, and, secondly, the reduction of healthcare costs thanks to these efficient and affordable (both in use and in economics) technologies [1,2,3,4,17,18,28,29].

WBS are a specific category of PHS. They are integrated systems that can be embedded into a wearable platform (sometimes and optimally in the sense of clothing) or a net of devices attachable to the human body for continuous monitoring of biomedical, biochemical, and/or physical parameters. In this way, it is possible to achieve early detection of anomalies. This patient awareness can produce an increased feeling of safety and confidence, which is perceived as an increase in quality of life. In addition, this kind of ambulatory monitoring can allow patients to engage in normal activities of daily life, rather than staying at home or close to specialized medical services. Thus, WBS represent an extraordinary opportunity to support the provision of a remote primary and secondary prevention, to obtain early diagnosis and management of several diseases (in particular, cardiovascular and/or respiratory diseases, metabolic pathologies, and the assessments of physical rehabilitation treatments), and to support elderly and disabled people.

WBS can be used to measure a plurality of signals from the human body; the main and most frequently used ones are heart rate (or even ECG tracks), electro-encephalography, respiration, blood gases saturation, and body movements. They represent a good balance between system complexity and user compliance for a light or general monitoring of the health status through the following main physiological parameters. A good wearable device for monitoring could include 1 or 2 ECG leads, breathing parameters (respiratory rate but also in/expiration times and volumes), and should accommodate human activity and posture (through one or more three-axial accelerometers or IMUs).

**Figure 8:**
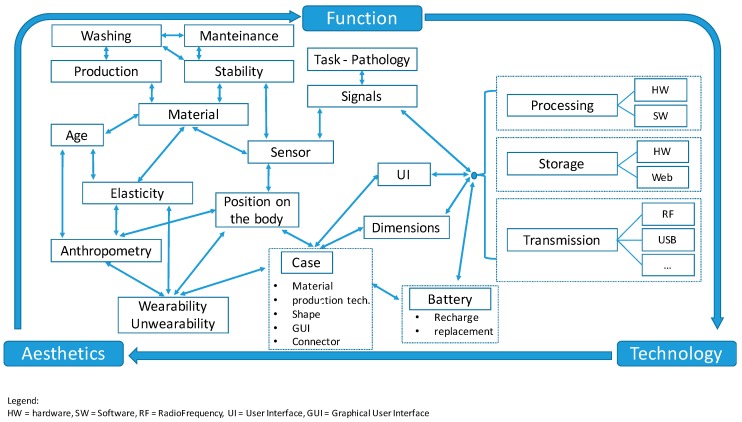
A methodology and the corresponding decision tree for designing smart sensing garments.

From the results that we have presented and discussed in this paper, in designing smart sensing garments and WHS, we can identify three main requirements: aesthetics, function, and technology. They do not stand alone; they are strongly interdependent. When facing a new project, the basic questions are the task or pathology to be used, and from this first aspect, one must identify the signal of interest and the corresponding sensors. Then, the technological choices and requirements are to be defined accordingly. At the same time, from the identification of the task or pathology, the aesthetical requirements for wearability and elasticity descend. These also influence the definition of shapes, body position, and size of the WHS in strong correlation with the technological choices. This decision tree is shown in Figure 8.

The most frequently used sensors for bioelectric signals (e.g., ECG, EMG, and EEG) are the surface electrodes, whose textile version can be integrated into garments to replace the standard adhesive silver/silver chloride (Ag/AgCl) electrodes, which are not suitable for long-term continuous monitoring because of skin irritation problems that are shown in a relevant number of subjects.

Usually, the last issue to be considered when designing WHS is the connection between the electronic device and the textile component (the garment or the supporting element). The essential requirement is the presence of a constant, stable, and rigid link between the two parts (specifically for IMUs attached to the body to monitor movements). This will ensure the possibility of obtaining a good signal quality during its recording. If the number of contacts is low (2 or 3 maximum) so that standard pins can only produce a weak link, a specific design of the device including a connecting snap on its case can be achieved. In this way, a connection through stainless steel nickel free snap buttons could be provided to solve this issue. However, if the number of required contacts is higher (five or more), the snaps solution becomes difficult to adopt because of the low acceptance expressed by users; therefore, a specifically designed or dedicated connector, possibly integrating flexible PCB or flexible/rigid PCB technology, could be the optimal choice to deal with this complexity in matching components. In fact, even if this is an expensive solution, it still represents the best way to optimize the interface between textile and hardware components without compromising the flexibility and easy wearability of the overall system.

As a result of the previous discussion, a basic methodological approach can be proposed. The process of designing a wearable monitoring system can be described as the balance of three elements: function, technology, and aesthetics. They are strongly interconnected and interdependent. The design path and related choices can start from each point; however, the definition of the system’s specification should follow the identification of different components. They are summarized and represented in Figure 8.

The function requirements or specifications are related to monitoring needs; the basic requirements are:

- human parameters to be monitored and related signals for the application or pathology, identifying the main signal and computed parameters;
- typology and numbers of sensors required for the monitoring;
- a monitoring paradigm—continuous signal transmission or delayed data transfer;
- real-time data processing and analysis or off-line operations, secondary parameter computation and storage on-board or remote solutions;
- direct patient participation or other caregivers operations, and, indirectly but obviously, who the subjects to be monitored are and their features (age, sex, body build, *etc.*), which are also pieces of information very relevant for aesthetical specifications. Secondary function requirements are related to the production and maintenance of the sensors and the devices.

Moving towards human parameters, the identification of the signals and their related functions drives the choice of the sensors and the preferable position on the body; at the same time, it gives the essential requirements for hardware and software design or selection. In the case of the design of the technological solution, this will also match the needs in terms of shape, dimensions, and position on the body. User Interfaces (UIs) are the physical interfaces between the device and the user (buttons, switches, fixing supports, *etc.*) or the sensors (connectors) and provide input to garment design and system usability. If standard or commercial devices or systems are to be adopted, the technological features only apply to garment design for connection with the embedded sensors or to the building of the proper supports for the device itself (pockets or rigid element for fixing the system to the body in respect of the maximization of the non-intrusive monitoring approach).

The garment design relates to the selection of materials (type of yarns and related production techniques) as well as design and customization of the sensing textile, also in consideration of wearability and un-wearability. Motor disorders or age can influence the freedom of movement or specific needs for these tasks, thus determining the acceptance of the solution. The system usability is linked to the wearability but also implies other features such as GUI and maintenance. Elasticity and adherence to the body and minimization of the sensors displacement over the skin is crucial, both from the technological point of view and for the ergonomics of the garment. In designing the garment, other secondary but relevant issues to be considered are:

- integration of textile wiring through conductive yarns connecting the sensor to the measuring device;
- sweat and thermal management to assure a good quality and then garment acceptance to the users;
- specific requirements for washing, ironing, and sterilization or disinfection should be considered if applicable in special applications (e.g., monitoring in an Intensive Care Unit or in the case of the presence of exposed or open wounds).

This design paradigm is just an example. The design path could follow different directions following the different inter-relations described in Figure 8. The important conclusion is to face all the requirements and to make the proper decisions.

A similar approach could also be imagined in the case of wearable devices not including a textile component, but a simple device worn on a specific body part (e.g., as a bracelet, necklace, earring, shoe insole, *etc.*).

## 4. Conclusions

In this paper we have presented some issues related to the design of WBS systems with specific reference to requirements related to technology and ergonomic or human factors. However, this method is not a rule. It is a proposal deriving from our experiences in designing smart sensing garment both medical applications (in monitoring mother and fetus in pregnancy, in preterm babies or newborn in the first two hours of life, in cardiologic adults, in elderly at home or in hospital during rehabilitation exercises) and in sport activities (adolescents in their physical education at school or monitoring their lifestyle during the whole day, adults while running, playing soccer and skyrace) [6,7,8,10,11,12,13,14,17,18,20,21,22,23,28,29]. In this process, co-design activities are proposed and carried out for better matching user preferences and aesthetics requirements that significantly vary in relation to age and human anthropometry.

Wearable systems are rapidly spreading internationally because they are easy-to-use. They use our natural interface with respect to the external world to integrate sensing or actuation capabilities. This fact is crucial because it allows for the actual implementation of the non-intrusive monitoring paradigm, *i.e.*, a process that does not affect user behavior and is completely transparent to him/her during his/her own daily activities. Only this methodological approach to be pursued by wearable systems can lead to the innovative wearable 2.0 generation systems.

We expect that this development and diffusion will continue in the next 5 to 10 years and then will become stable because the new generations of garments will directly include these features. Lifestyle monitoring and light medical monitoring are becoming essential for the new trends in healthcare and wellness programs. However, long and hard work is still necessary to exploit the sector and also work through legal/privacy issues for services or data management that have to be properly regulated.

Sport is another very important and huge market: performance and training analysis together with injury prevention or optimization for the functional recovery after it, are activities supported in a very promising way by wearables.

At the same time, we cannot forget that a new frontier is arising: the “insideable” systems. Electronics miniaturization and new biocompatible materials are opening this innovative era. However, this will be another story.

## Figures and Tables

**Table 1:** The basic signals and the derived parameters to be measured by wearable sensing systems, and the identification of the sensor to be used and its possible typologies. ECG: Electro-Cardio-Gram, EMG: Electro-Myo-Gram, EEG: Electro-Encephalo-Gram.

No.	Signal ^1^	Parameter (s)	Sensor	Typology
1	ECG	Electrical heart activity, Heart Rate	electrode	Adhesive, textile, plate
2	EEG	Electrical Brain activity	electrode	Plate, textile prototypes
3	EMG	Electrical muscle activity	electrode	Adhesive, textile prototypes
4	Respiration	Breathing rate, Volumes, respiratory times	Strain gauge; Electrode for impedance measure	Hardware probe, adhesive, textile sensor
5	Blood gas	SpO_2_, CO_2_, Heart rate	LED/optical	Hardware probe, POF for signal transmission
6	Blood pressure	Systolic/diastolic values, Heart rate	Cuff	Hardware System
7	Interface pressure	Contact pressure	Piezoresistive; capacitive	Piezoresistive ink, capacitive sensor both electric and textile
8	Resistance	GSR, Body impedance	Electrodes for Impedance measure	Hardware System
9	Temperature	Temperature	Piezoresistive	Hardware probe (thermistor)

^1^ Note: Movement is measured with miniaturized hardware system applied to each body district.

**Table 2:** Materials for textile electrodes and related properties.

Material	Merits	Demerits
Conductive rubber	High conductivity, easy to shape, cheap	Poor flexibility and permeability to air and liquid
Silver-coated polymer foam	High conductivity, easy to shape, flexible, antibacterial	Poor washability and permeability to air and liquid, possible oxidation
Metal-coated or sputtered fabric	High conductivity, fabric material	Poor washability, possible oxidation
Woven metal fabric	Controlled conductivity, fabric material	Difficult to handle, skin irritation, low elasticity
Woven conductive polymer fabric	Fabric material, elasticity	Low conductivity
Carbon yarn	High mechanical resistance, high thermal insulation	Average conductivity, skin irritation, low elasticity
Stainless steel yarn	High conductivity, no skin interaction	Low elasticity, high weight

## Abbreviations

ECG	Electro-Cardio-Gram
EEG	Electro-Encephalo-Gram
EMG	Electro-Myo-Gram
ENG	Electro-Neuro-Gram
EOG	Electro-Oculo-Gram
GUI	Graphical User Interface
IMU	Inertial Movement Unit
NIRS	Near Infra-Red Spectroscopy
PCB	Printed Circuit Board
PHS	Personal Health Systems
POF	Plastic Optical Fiber
SpO_2_	Peripheral Capillary Oxygen Saturation
UI	User Interface
WBS	Wearable Biomedical Systems
WHS	Wearable Health Systems
